# RalA, a GTPase targeted by miR-181a, promotes transformation and progression by activating the Ras-related signaling pathway in chronic myelogenous leukemia

**DOI:** 10.18632/oncotarget.7987

**Published:** 2016-03-08

**Authors:** Chunming Gu, Maoxiao Feng, Zhao Yin, Xiaochuang Luo, Juhua Yang, Yumin Li, Tianfu Li, Ruirui Wang, Jia Fei

**Affiliations:** ^1^ Department of Biochemistry and Molecular Biology, Medical College of Jinan University, Guangzhou 510632, China; ^2^ Insititute of Chinese Integrative Medicine, Medical College of Jinan University, Guangzhou 510632, China; ^3^ Department of Clinical Medicine, Medical College of Jinan University, Guangzhou 510632, China

**Keywords:** RalA GTPases, chronic myelogenous leukemia, malignant transformation, imatinib, Ras signaling pathway

## Abstract

BCR/ABL is a well-known activator of multiple signaling pathways. RalA, a Ras downstream signaling molecule and a small GTPase, plays an important role in Bcr-Abl-induced leukemogenesis but the exact mechanism remains elusive. Here, we show that RalA GTPase activity is commonly high in chronic myelogenous leukemia (CML) cell lines and patient samples. Overexpression of RalA results in malignant transformation and progression, and induces resistance to imatinib (IM) in BaF3 and K562 cell lines. RalA reduced survival and led to IM resistance in a xenografted mouse model. Ablation of RalA by either siRNA or miR-181a, a RalA targeting microRNA, attenuated the malignant phenotypes in K562 cells. RBC8, a selective Ral inhibitor, enhanced the inhibitory effects of IM in K562, KCL22 and BaF3-P210 cells. Interestingly, the phospho-specific protein microarray assay revealed that multiple phosphorylation signal proteins were decreased by RalA inhibition, including SAPK, JNK, SRC, VEGFR2, P38 MAPK, c-Kit, JunB, and Keratin18. Among them, P38 MAPK and SAPK/JNK are Ras downstream signaling kinases. Taken together, RalA GTPase might be an important oncogene activating the Ras-related signaling pathway in CML.

## INTRODUCTION

Chronic myelogenous leukemia (CML) is a malignant disorder of hematopoietic stem cells that arises from reciprocal translocation between the *BCR* gene on chromosome 22 and the *ABL* gene on chromosome 9, t (9;22) (q34;q11), called Philadelphia chromosome [1, 2]. The enhanced tyrosine kinase (TK) activity of BCR/ABL plays a critical role in hematopoietic cell transformation in CML. Imatinib mesylate (IM), a small molecule tyrosine kinase inhibitor (TKI) that binds to the ATP-binding site of ABL and inhibits BCR-ABL kinase activity, has proven to be a revolutionary treatment for patients with CML [3–5].

Despite impressive clinical responses, 20–30% of patients treated with IM fail to achieve a complete cytogenetic response. Patients with optimal responses may also subsequently relapse. Moreover, approximately 15% of IM-treated patients only achieve suboptimal and temporary responses that require higher doses of IM or changes in drug combinations [4]. IM resistance is also common. Several mechanisms have been shown to contribute to such resistance, including ABL kinase domain mutation, BCR/ABL protein overexpression, and an increase in P-glycoprotein or other oncogenes [6]. In ∼20% of CML cases, TKI resistance is not caused by altered BCR/ABL function. However, this BCR/ABL-independent IM resistance is not well understood [7, 8]. Also, it is conceivable that drugs targeting alternative signal transduction pathways and synergizing with IM may enhance the efficacy of targeted therapies. Therefore, molecular targets of BCR/ABL downstream may be attractive candidates for CML treatment. For example, the Ras pathway is activated by BCR/ABL and plays a key role in BCR/ABL-controlled leukemogenesis [9, 10]. Thus, inhibiting the Ras signaling pathway might be a prospective strategy for overcoming IM resistance in CML.

Recent studies have demonstrated that some Ras effector molecules, such as Rac GTPases, CDC42 GTPase and RhoA GTPase, play a crucial role in Bcr-Abl-induced leukemogenesis [11–14]. RalA GTPase, a member of the Ras effectors, has been implicated in tumorigenesis, invasion, and metastasis of a variety of solid tumors [15]. Thus, activation of RalA signaling appears to be a critical step in Ras-induced transformation and tumorigenesis [16]. RBC8 and BQU57, selective Ral inhibitors, can inhibit xenografted tumor growth (bladder cancer cell line and the human lung cancer cell lines) [17]. However, the exact role of RalA in CML remains elusive.

We have shown previously that RalA is a direct target of miR-181a and plays an important oncogenic role in CML [18, 19]. These findings provide a new mechanistic insight into the role of RalA GTPase in CML and suggest that RalA could be a potential target for therapeutic intervention in CML and possibly other cancers as well.

## RESULTS

### Increased RalA GTPase activity increases malignant transformation in CML cells

**Figure 1:**
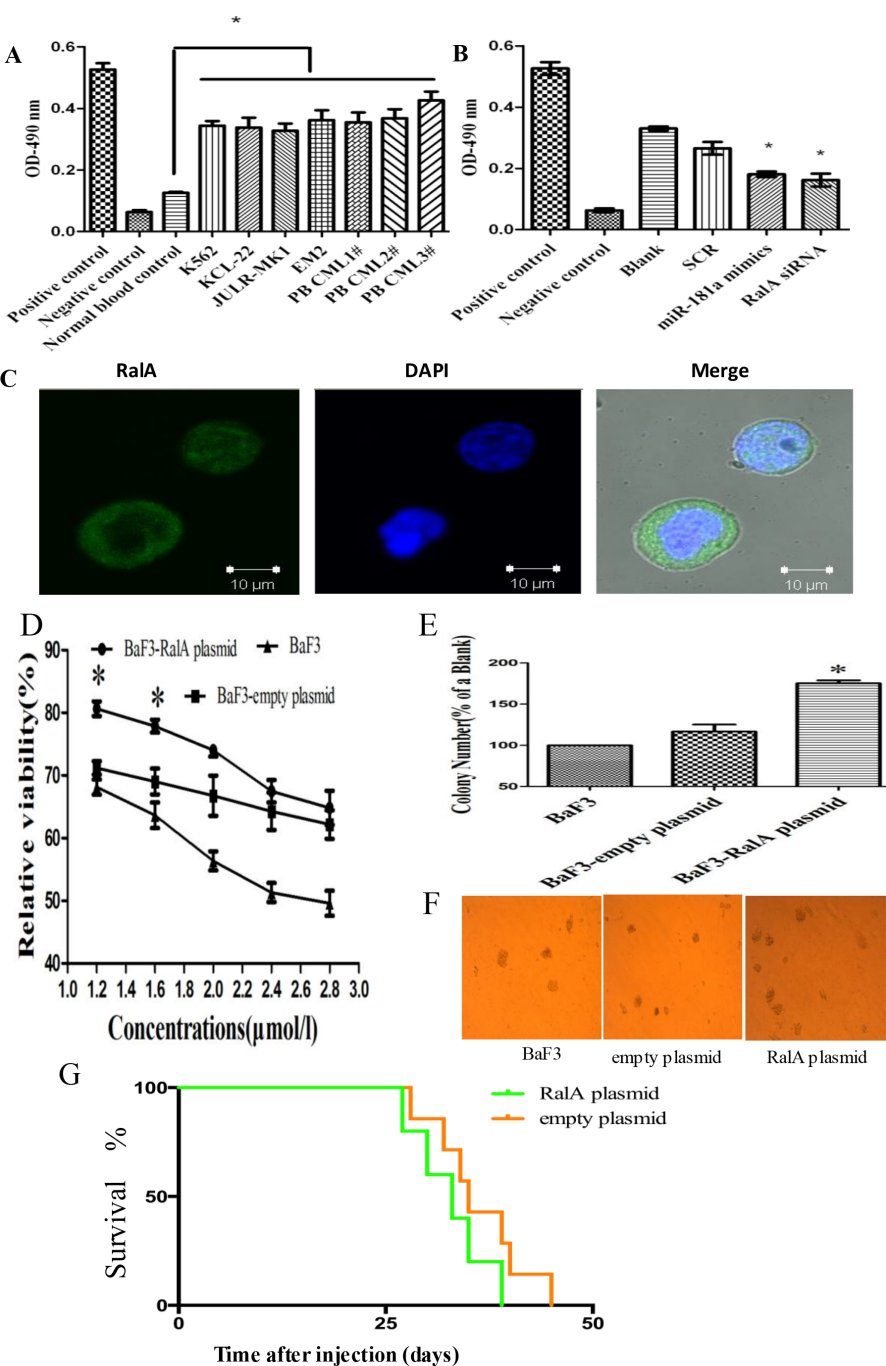
RalA GTPase activity is increased in CML cells (**A**) RalA GTPase activity was determined by G-LISA^™^ in CML cell lines and three CML peripheral blood samples, compared to normal healthy blood controls. (**B**) RalA GTPase activity measured by G-LISA^™^ in K562 cells with overexpression of miR-181a or RalA siRNA compared to scrambled controls (SCR). (**C**) Confocal microscopy analysis of the Ral A localization in K562 cells. (**D**) BaF3 cells transfected with RalA plasmid, empty vector or blank were treated with different concentrations of Ara-C (1.2–2.8 μM) for 48 h and the viability of cells was determined by MTT assay. The preliminary experiment showed that overexpression of RalA could counteract Ara-C-induced cytotoxicity, and increase colony formation capacity in BaF3 cells. (**E** and **F**) 1000 BaF3 cells with RalA vector were mixed with RPMI-1640 medium containing 0.9% methylcellulose solution and 20% FBS, and seeded onto 24-well plates. Colony numbers were counted after 1 week. Histogram (E) shows the relative number of colonies per 1000 plated cells. Overexpression of RalA increases the colony-forming activity in BaF3 cells. (**G**) Xenograft model of CML with the human cell line K562 with RalA or empty vector in Balb/c mouse. 500,000 K562 cells transfected with RalA or empty vector were injected via intravenous tail vein injection into sublethally irradiated Balb/c recipient mice. Overall survival is shown by Kaplan–Meier analysis.

We have previously shown that miR-181a directly targets RalA GTPase in CML cells [18, 19]. However, the role of RalA in CML is poorly understood. RalA GTPase becomes activated when it switches from the GDP-bound state to the GTP-bound state that specifically interacts with their downstream effector proteins. To further understand the role of RalA in CML, we first tested the expression and activity of RalA in four CML cell lines and three primary CML samples. As shown in Figure 1A, RalA GTPase activity was significantly higher in these CML cells compared to normal blood control. Consistently, RalA GTPase activity was significantly decreased upon inhibition of RalA expression with either overexpression of miR-181a mimic or RalA siRNA (Figure 1B). These results indicate that RalA GTPase activity is increased in CML cells and thus may be a new biomarker. Confocal immunohistochemistry showed that RalA protein was located mainly in the cytoplasm in K562 cells (Figure 1C). Our preliminary studies show that overexpression of RalA counteracts Ara-C-induced cytotoxicity (Figure 1D) and increases colony formation capacity in BaF3 cells (Figure 1E and 1F), indicating that RalA may promote transformation of murine immortalized normal pro-B BaF3 cells. In mice xenografted with K562 cells, all of these animals had developed varying symptoms of CML, i.e. cachexia, apathy, palpable tumors/chloromas. Overall survival, and leukemia progression were monitored by Kaplan–Meier analysis. The results showed that overexpression of RalA reduced overall survival of recipient mice (Figure 1G).

### RalA GTPase promotes malignant progression of CML cells

**Figure 2:**
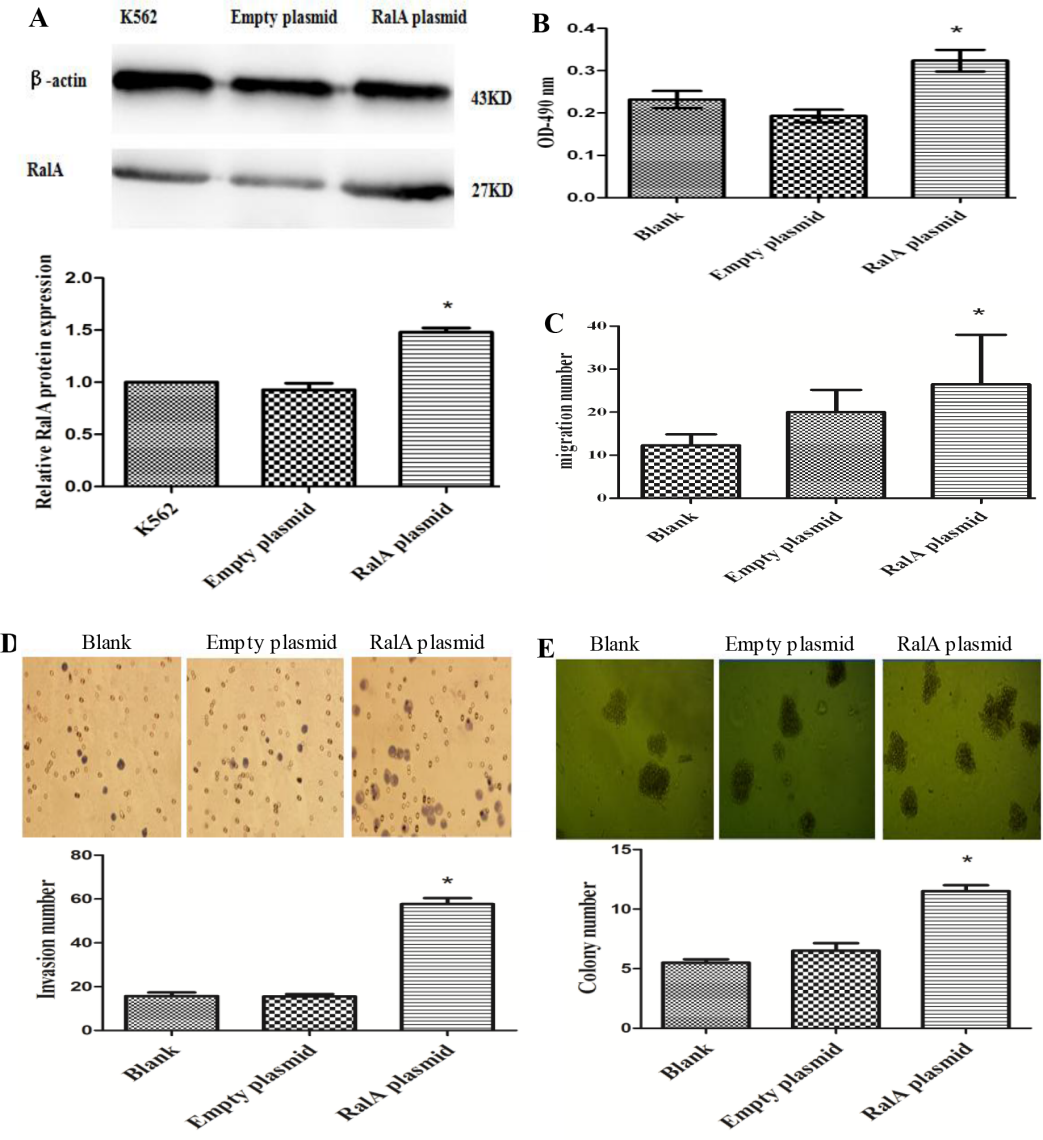
High RalA GTPase activity increases K562 cell malignant progression (**A**) Expression of exogenous RalA in K562 cells. K562 cells transfected with RalA or empty vector were analyzed for the expression of RalA by immunoblot (IB). (**B**) The RalA activity of cells transfected with RalA plasmid was measured by G-LISA^™^. (**C**) Overexpression of RalA promotes migration in K562 cells. A total of 6 × 10^5^ cells transfected with control or RalA plasmids were seeded into the upper chambers, followed by counting of the cells in the lower chambers after 24 h. (**D**) Overexpression of RalA promotes invasion of K562 cells. A total of 3 × 10^5^ cells transfected with control or RalA plasmids were seeded into the upper chambers coated with Matrigel. Cells in the lower chambers were stained with hematoxylin and counted after 8 h. Histogram and statistics showing the relative invasion rate are shown in the lower panel (left). (**E**) Overexpression of RalA increases the colony formation of K562 cells. A total of 1000 cells transfected with control or RalA plasmids were mixed with RPMI-1640 medium containing 0.9% methylcellulose solution and 20% FBS, and seeded onto 24-well plates. Colony numbers were counted after 1 week. Histogram and statistics indicating the relative number of colonies per 1000 plated cells are shown in the lower panel (right). Statistical significance was assessed by one-way ANOVA (*p < 0.05).

To test whether the increased RalA GTPase activity contributes to cell malignant phenotypes in CML cells, we constructed a RalA expression plasmid for RalA overexpression studies. As shown in Figure 2A and 2B, the expression of RalA protein (Figure 2A) and activity (Figure 2B) were significantly increased in K562 cells transfected with the RalA expression plasmid compared to empty vector transfected cells. We then conducted cell migration and invasion assays using the Transwell assay in the absence or presence of BD Matrigel, respectively. Cell migration and invasion are key malignant behaviors that are associated with tumorigenesis and cancer metastasis. As shown in Figure 2C and 2D, overexpression of RalA significantly promoted cell migration and invasion, respectively (*P* < 0.05). Colony-forming assays were also performed to measure cell neoplastic capacity. As shown in Figure 2E, overexpression of RalA significantly increased the number of colonies in K562 cells transfected with RalA, compared to the empty vector transfection. Together, these results indicate that increased levels and GTPase activity of RalA promote malignant phenotypes in K562 cells.

### Overexpression of RalA in K562 cells confers IM resistance

**Figure 3:**
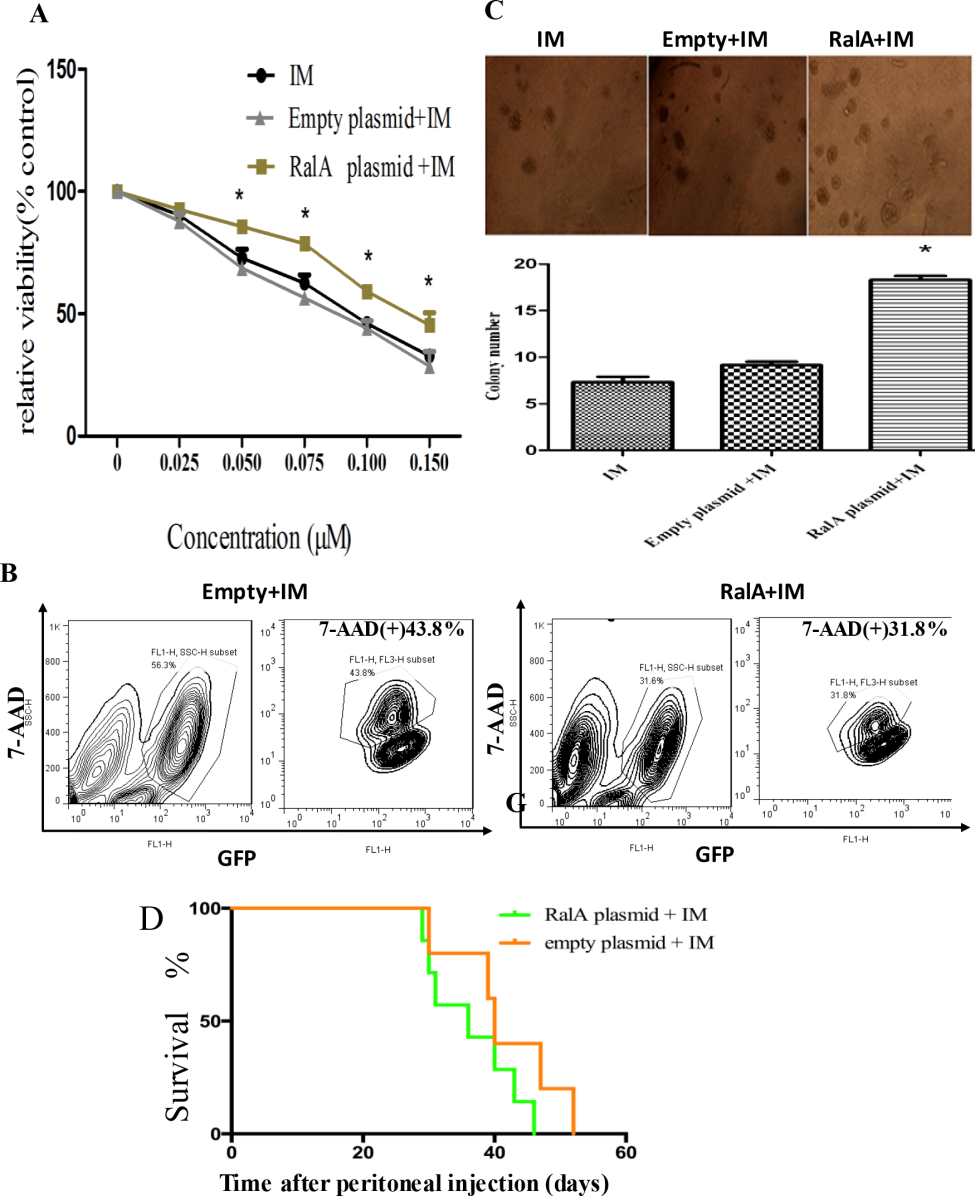
Overexpression of RalA in K562 cells confers IM resistance (**A**) K562 cells transfected with RalA plasmid, empty vector or blank were treated with different concentrations of imatinib (0–0.15 μM) for 48 h, followed by MTT assays. (**B**) Overexpression of RalA reduces apoptosis following IM treatment. K562 cells transfected with RalA plasmid, empty vector or blank were treated with IM (0.1 μΜ) for 48 h. The cells were washed and stained with 7-AAD and then analyzed by flow cytometry to determine apoptosis. Statistical significance was assessed by one-way ANOVA (*p < 0.05). (**C**) Colony formation assays. A total of 1000 cells transfected without or with control or RalA plasmids were mixed with RPMI-1640 medium containing 0.9% methylcellulose solution, 20% FBS and IM (0.75 μΜ), and seeded onto 24-well plates. Colony numbers were counted after 1 week. (**D**). Xenograft model of CML with the human cell line K562 with RalA or empty vector in Balb/c mice. 500,000 K562 cells transfected with RalA or empty vector were injected via intravenous tail vein injection into sublethally irradiated (3 Gy) Balb/c recipient mice. Imatinib was injected intraperitoneally at a dose of 100 mg/kg body weight for 10 consecutive days. Overall survival is shown by Kaplan–Meier analysis.

To test whether the high expression of RalA in CML relates to therapeutic response, IM sensitivity was analyzed in RalA-overexpressing K562 cells by MTT and FACS analysis. Cells transfected with control or RalA expressing plasmids were treated with IM for 24 h. The results showed that overexpression of RalA significantly inhibited the cell killing effect of IM in K562 cells as indicated by increased cell viability (Figure 3A), and reduced cell death as indicated by positive 7-AAD staining (Figure 3B). Furthermore, in the presence of IM, K562 cells transfected with RalA plasmid formed more colonies in culture (Figure 3C). These results indicate that overexpression of RalA was sufficient to render K562 cells resistant to cell death induced by IM. Overall survival was monitored by Kaplan–Meier analysis. The results showed that overexpression of RalA reduced overall survival of recipient mice undergoing IM treatment, indicating that RalA imparts some resistance to IM (Figure 3D).

### Targeted inhibition of RalA attenuates the malignant properties of CML cells

**Figure 4:**
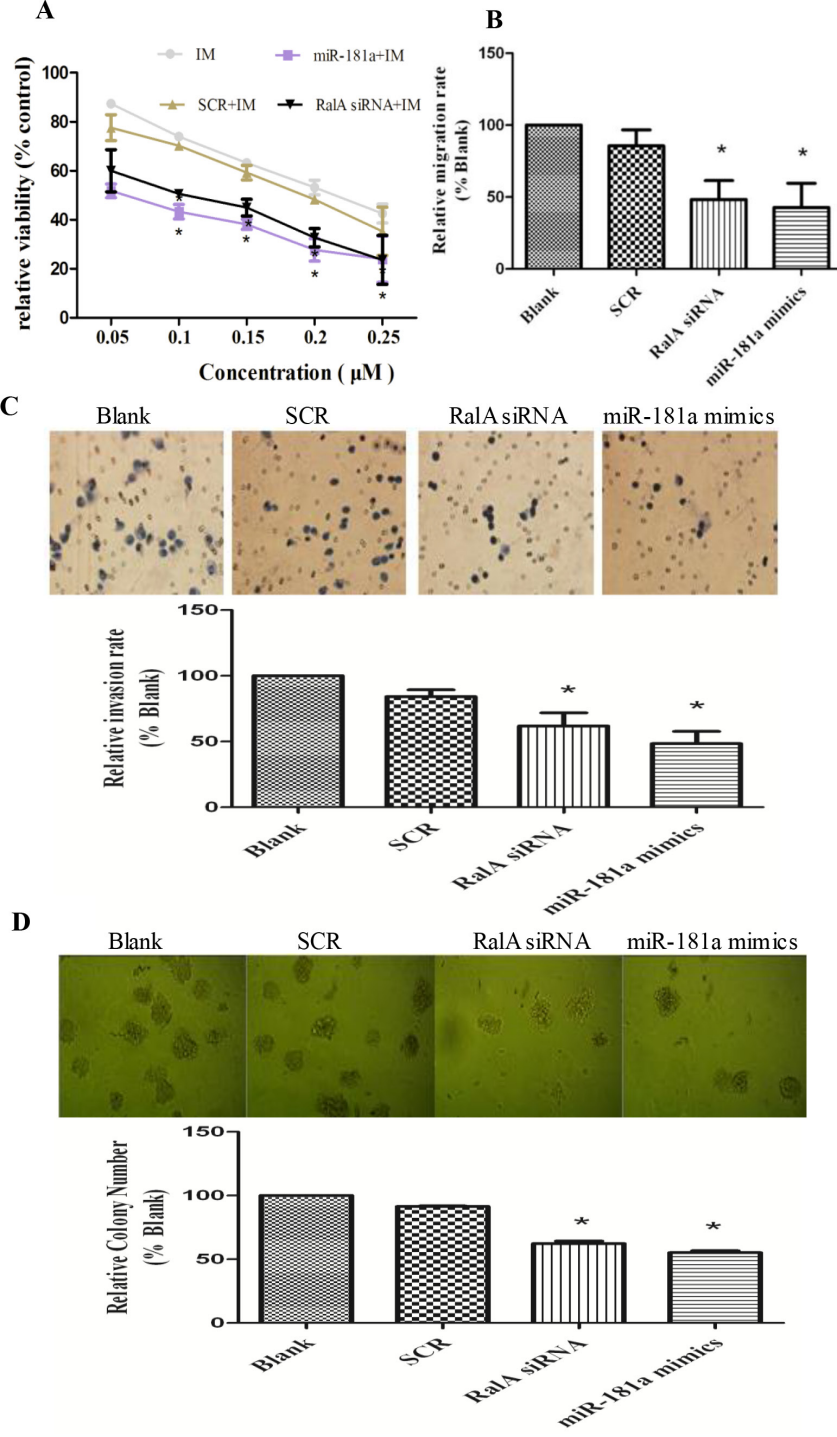
Targeted inhibition of RalA attenuates CML malignant progression and sensitizes K562 cells to imatinib treatment (**A**) Inhibition of RalA sensitizes K562 cells to imatinib. K562 cells transfected with control, RalA siRNA or miR-181a were treated with different concentrations of imatinib (0–0.25 μM) for 24 h and the viability of cells was determined by MTT assay. (**B**) Inhibition of RalA reduces CML cell migration. A total of 6 × 10^5^ K562 cells transfected with control, RalA siRNA or miR-181a were seeded into the upper chambers, counted after 24 h, followed by counting of cell numbers in the lower chambers. (**C**) Inhibition of RalA reduces CML cell invasion. A total of 3 × 10^5^ cells transfected with control, RalA siRNA, or miR-181a were seeded into the upper chambers coated with Matrigel, stained with hematoxylin and counted after 8 h. (**D**) Inhibition of RalA reduces K562 cell colony formation. A total of 1000 K562 cells transfected with control, RalA siRNA, or miR-181a were mixed with RPMI-1640 medium containing 0.9% methylcellulose solution and 20% FBS, and seeded onto 24-well plates. Colony numbers were counted after 1 week. Histogram and statistics indicating the relative number of colonies per 1000 plated cells are shown in the lower panel. Statistical significance was assessed by one-way ANOVA (*p < 0.05).

Next, we studied whether reducing RalA GTPase activity by either RalA siRNA or miR-181a, which target RalA, could attenuate CML malignant behavior. As shown in Figure 4A, increasing concentrations of either miR-181a or RalA siRNA effectively increased K562 cell sensitivity to IM treatment. Transwell assays with or without Matrigel also showed that the migration and invasion ability of CML cells were significantly reduced by miR-181a mimic or RalA siRNA (Figure 4B and 4C). Finally, the colony-forming ability of K562 cells transfected with miR-181a or RalA siRNA was significantly reduced compared to cells transfected with scrambled RNA control (*P* < 0.05) (Figure 4D). Taken together, these data demonstrate that targeted inhibition of RalA attenuates CML malignant properties.

### RBC8 inhibits cell viability and has a synergic effect with imatinib

**Figure 5:**
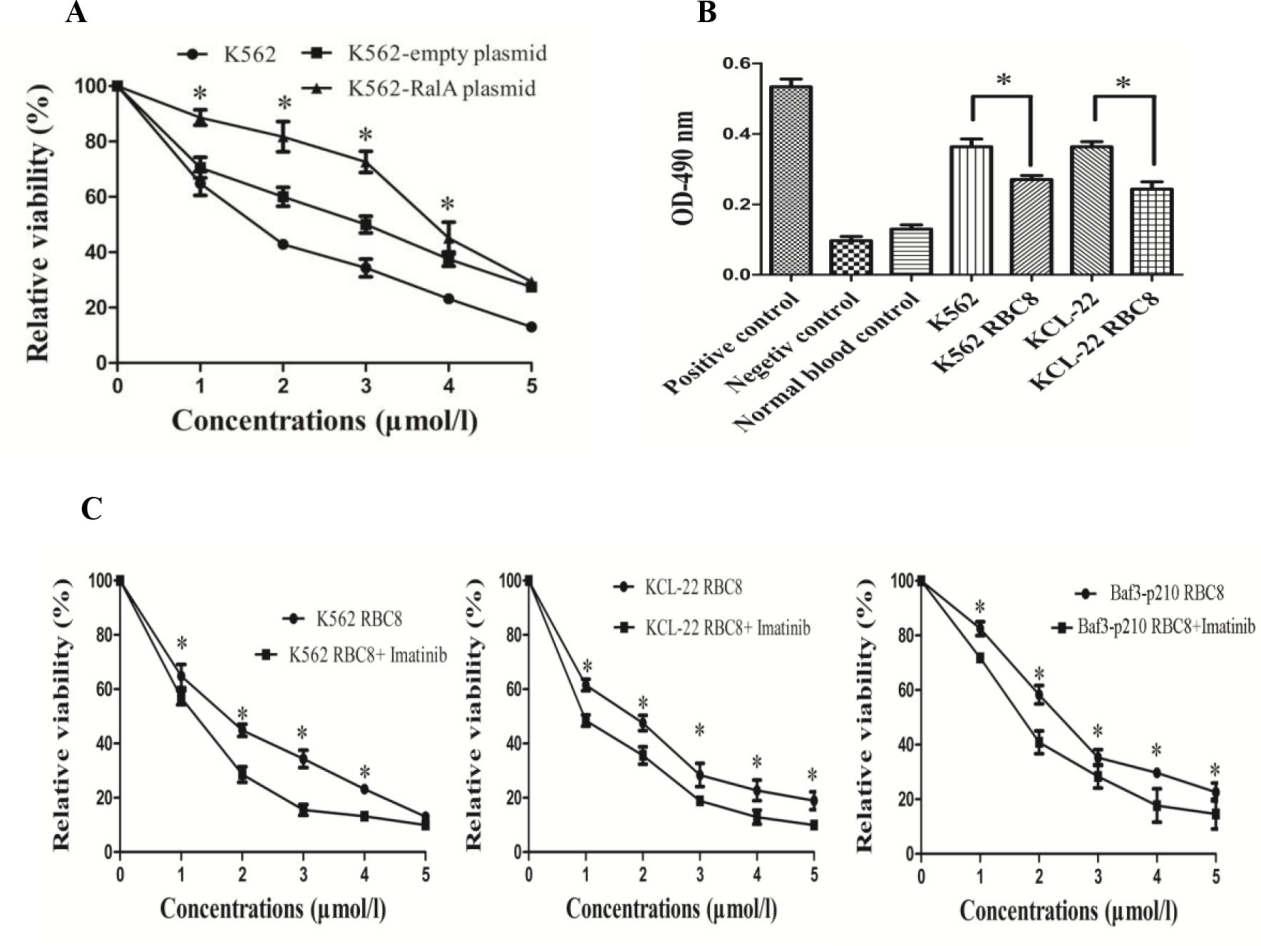
RBC8 inhibits cell viability and their synergic effects with imatinib (**A**) RBC8 inhibits the viability of K562 cells. K562 cells transfected with RalA plasmid, empty vector or blank were treated with different concentrations of RBC8 (1–5 μM) for 48 h, followed by MTT assays. (**B**) RalA GTPase activity measured by G-LISA^™^ in K562 and KCL-22 cells compared to normal blood controls. K562 and KCL-22 cells were treated with RBC8 (3 μM) for 48 h, followed by G-LISA^™^ assays. (**C**) RBC8 enhances the inhibitory effects of imatinib. K562, KCL-22 and BaF3-P210 cells were treated with different concentrations of RBC8 (1–5 μM) plus imatinib (0.1 μM) for 48 h, followed by MTT assays.

RBC8 is a selective inhibitor of RalA GTPases. Here, we studied the inhibitory effects of RBC8 alone and in combination with imatinib. The results showed that RBC8 effectively inhibited K562 cell viability, and overexpression of RalA could produce some resistance to RBC8 (Figure 5A). Meanwhile, RBC8 significantly reduced RalA GTPase activity in K562 and KCL22 cells (Figure 5B). Furthermore, RBC8 enhances the inhibitory effects of imatinib in K562, KCL22 and BaF3-P210 cells (Figure 5C). Overall, RBC8 targeting alternative RalA signal pathways and synergizing with imatinib may enhance the efficacy of targeted therapies.

### RalA GTPase decreases Ras downstream phosphorylation

**Figure 6:**
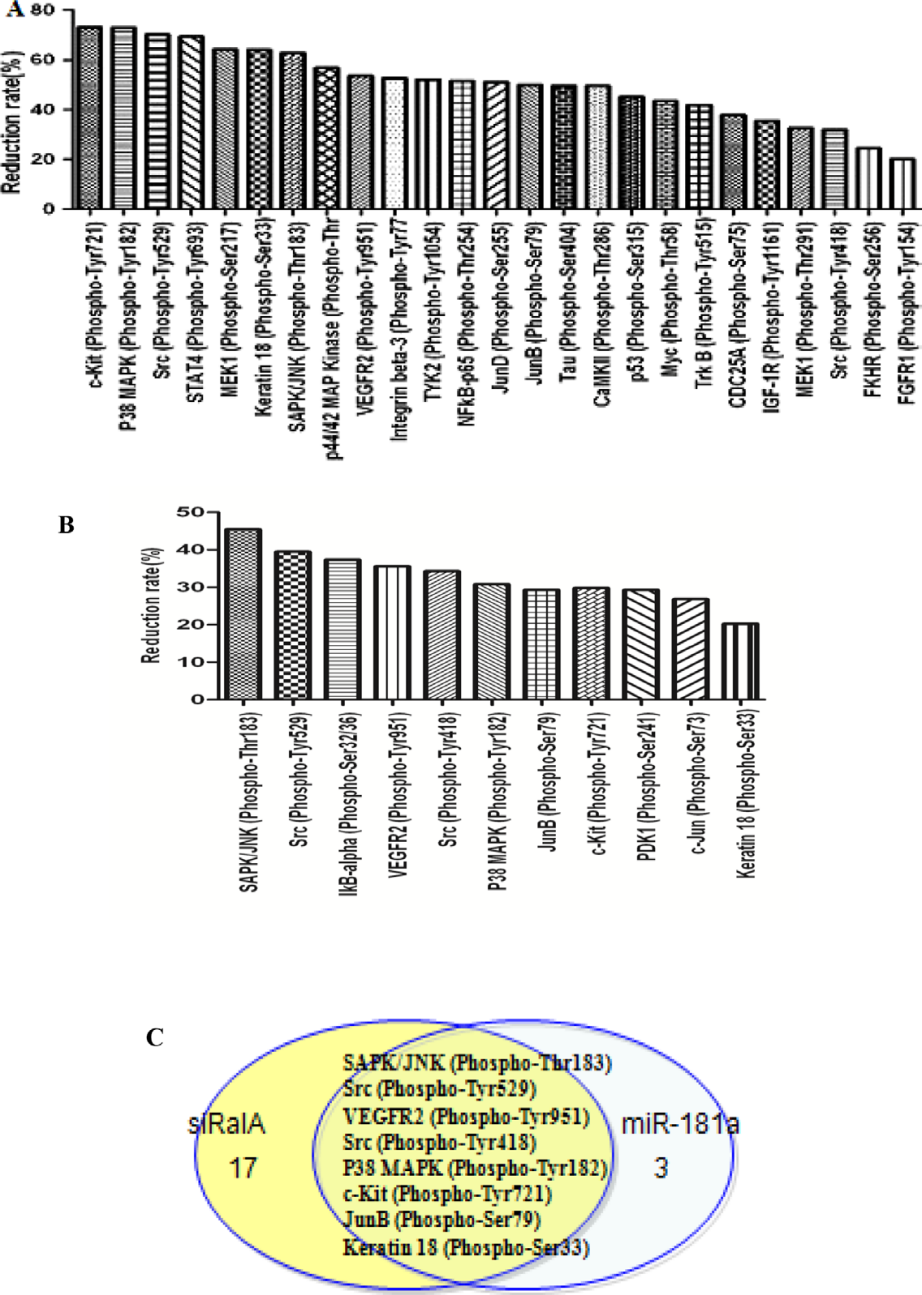
Inhibition of RalA by RalA siRNA or miR-181a mimic reduces the phosphorylative signal proteins Phosphorylative signal proteins in K562 cells transfected with RalA siRNA (**A**) or miR-181a (**B**) were comprehensively investigated using an antibody microarray system compared to control. The phosphorylation ratio (Phos Ratio) was used as the modulation difference of phosphorylation sites between two samples and was computed as follows: Reduction rate (%) = (Average value without RalA siRNA or miR-181a treatment–Average value with control treatment) / Average value with control treatment × 100. (**C**) Comparison of inhibitory spectrum of miR-181a and RalA siRNA results in the identification of phosphorylation targets that are associated with inhibitory effects of miR-181a and RalA siRNA in K562. Eight phosphorylation signal molecules were generally decreased by either miR-181a or RalA siRNA in K562 cells.

To understand the mechanisms underlying the role of RalA in enhancing migration, invasion, and clonogenicity of CML cells, we performed phospho-array assays. K562 cells transfected with control, miR-181a mimic, or RalA siRNA were subjected to analysis of protein phosphorylation profiles. Phosphorylation ratio (Phos Ratio) was used as the modulation difference between two samples, such as miR-181a and control microRNA mimic, or RalA siRNA and scrambled RNA control, at the phosphorylation sites. Among 248-site-specific phosphorylation in Cancer Signaling, the phosphorylation levels of 121 proteins were differentially expressed. Transfection with RalA siRNA reduced the phosphorylation of 57 out of 121 proteins. Among these proteins, phosphorylation decreased by > 20% was observed in 25 proteins. Notably, the highest decrease in phosphorylation was observed in c-KIT Tyr721 and P38 MAPK Tyr 182 (Figure 6A). In miR-181 transfection cells, phosphorylation levels were reduced at 23 protein sites, and 11 of them were reduced by > 20% (Figure 6B). A comparison of the inhibitory spectra allowed us to discern eight phosphorylation targets whose phosphorylation was reduced commonly by miR-181a and RalA siRNA, including SAPK/JNK (Phospho-Thr183), Src (Phospho-Tyr529), VEGFR2 (Phospho-Tyr951), Src (Phospho-Tyr418), P38 MAPK (Phospho-Tyr182), JunB (Phospho-Ser79), and Keratin 18 (Phospho-Ser33) (Figure 6C).

**Figure 7:**
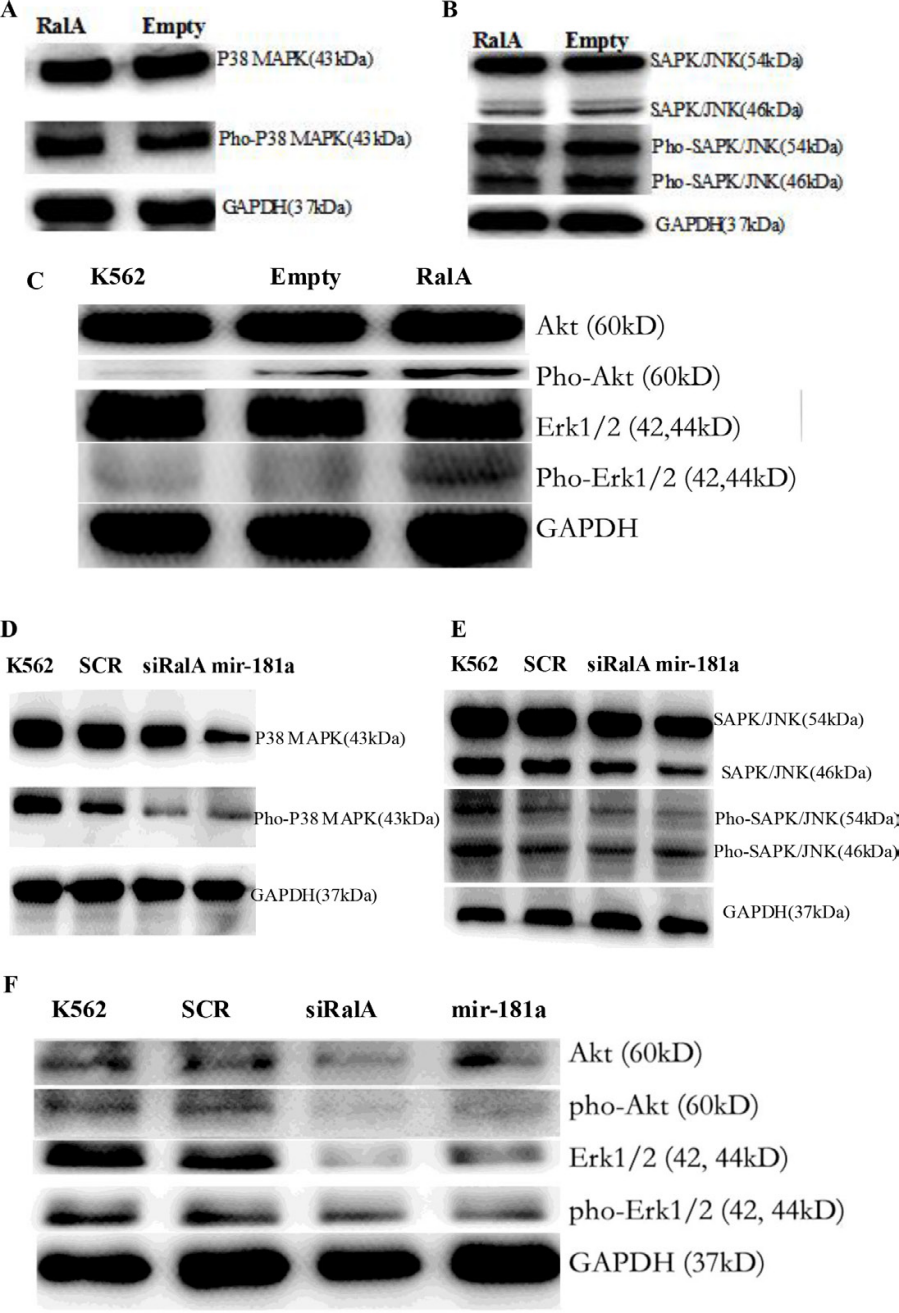
Western blot validation of the several key phosphorylation signal proteins through overexpression of RalA or RalA siRNA/miR-181a mimic Overexpression of RalA activation of Ras-related phosphorylative signaling pathway. Total cell lysate was isolated from K562 cells transfected with RalA plasmid, and analyzed for the expression levels of Phospho P38 MAPK (**A**), Phospho SAPK/JNK protein (**B**), and Phospho Akt/Erk (**C**). Inhibition of Ras-related phosphorylative signaling pathway by transfection with RalA siRNA or miR-181a. Total cell lysate was isolated from K562 cells transfected with RalA siRNA or miR-181a, and analyzed for the expression level of Phospho P38 MAPK (**D**), Phospho SAPK/JNK protein (**E**), and Phospho Akt/Erk (**F**).

It is known that P38 MAPK and SAPK/JNK are the downstream molecules in the Ras signaling pathway. Total cell lysate was isolated from K562 cells transfected with RalA siRNA and miR-181a, and analyzed for the Phospho-protein expression level. Overexpression of RalA could be activation of Ras-related phosphorylative signaling pathway. Total cell lysate was isolated from K562 cells transfected with RalA plasmid, and analyzed for the expression levels of Phospho P38 MAPK (Figure 7A), Phospho SAPK/JNK protein (Figure 7B), and Akt/Erk (Figure 7C). Western blot confirmed that RalA siRNA and miR-181a effectively inhibited the expression levels of Phospho P38 MAPK (Figure 7D), Phospho SAPK/JNK protein (Figure 7E), and Akt/Erk (Figure 7F).

KEGG pathway analysis of eight signal proteins (SAPK, JNK, SRC, VEGFR2, P38 MAPK, c-Kit, JunB, Keratin18) was studied. It was found that multiple signaling pathways (MAPK, ErbB, VEGF, mTOR, Wnt and PI3K-Akt) are closely correlated with CML pathogenesis and progression (Table S1). These results further suggest that RalA promotes cell invasion and clonogenicity by regulating the Ras signaling pathway in chronic myelogenous leukemia.

## DISCUSSION

In this study, we found evidence that RalA GTPase activity is significantly increased in CML cell lines and primary CML cells from patients compared to normal blood control (Figure 1A), without altering RalA expression in mRNA and protein levels (Figure S1). Confocal microscopy confirmed that RalA protein was located mainly in the cytoplasm or partially in the intracellular membrane in K562 cells (Figure 1C). These results imply that high RalA GTPase activity might be due to protein phosphorylation. Functionally, we showed that high RalA GTPase activity following RalA overexpression promotes CML cell migration, invasion, and colony formation, demonstrating the oncogenic activity of RalA (Figure 2).

Drug-resistance develops in a considerable proportion of CML patients and is the main impediment to prolonged survival. IM resistance often results from a secondary mutation in BCR-ABL that interferes with drug binding. Yet, in many instances, there is no mutation in BCR-ABL. Thus, the basis of such BCR-ABL-independent IM resistance remains to be elucidated [7, 8]. BCR/ABL is a well-known activator of multiple signaling pathways. We showed that high RalA GTPase activity attenuates IM-induced viability, colony formation and survival, thus producing an obvious resistance to IM (Figure 3). Consistently, targeted inhibition of RalA by either RalA siRNA or miR-181 attenuates CML malignant transformation (Figure 4).

RBC8 is a selective inhibitor of Ra1A GTPases by inhibiting RalA to its effector RALBP1, without inhibition of the Ras and RhoA GTPases [17]. We found that RBC8 could promote IM sensitivity (Figure 5). Therefore, RBC8 targeting of alternative RalA signal pathways and synergizing with IM may enhance the efficacy of targeted therapies.

miR-181 expression is commonly decreased in hematological malignancies. Meta-analyses have indicated that low miR-181a expression is significantly associated with poor survival outcomes in hematological malignancies [20, 21]. Also, altered miRNA expression, including miR-181, is involved in drug resistance in CML patients [22, 23]. This notion is further supported by our phospho-antibody microarray-based proteomic studies showing that phosphorylation and activation of multiple pro-metastatic proteins, downstream of RalA, were reduced by RalA inhibition in K562 cells. Among them, P38 MAPK and SAPK/JNK are ras downstream signaling kinases. Using KEGG pathway analysis, it was found that Ras activation of RalA leads to engagement of multiple effector pathways (MAPK, ErbB, VEGF, mTOR, Wnt and PI3K-Akt), which are closely correlated with CML pathogenesis and progression (Table S1). Western blot has confirmed the overexpression of RalA activation of Ras-related phosphorylative signaling protein, including Phospho P38 MAPK, Phospho SAPK/JNK, Phospho Akt and Phospho Erk (Figure 7A, 7B, 7C), whereas RalA siRNA and miR-181a inhibited the expression levels of these Phospho signaling molecules (Figure 7D, 7E, 7F).

We have previously shown that miR-181a expression is decreased, and miR-181a overexpression effectively suppresses cell growth and induces apoptosis in K562 cells [18]. We also showed that RalA is a direct target of miR-181a [18, 19]. Fredericks and Ren's study reported that the RalA pathway is important for rapid CML induction. Inhibiting this pathway via dominant negative RalA can delay disease progression [11]. This study also supports the notion that RalA inhibition is a potential therapy for BCR/ABL-induced CML.

In conclusion, our results indicate that high RalA GTPase activity results in malignant transformation and IM resistance in CML cells, and thus RalA could be a therapeutic target for CML.

## MATERIALS AND METHODS

### Cell lines, primary cells and cell culture

K562 CML cell line was obtained from the Institute of Shanghai Cell Biology, China. KCL-22, EM2 and Jurl-MK1 cells were kind gifts from Professor Markus Muschen, Children's Hospital of Los Angeles, CA, USA. BaF3 cells were kindly presented by Professor Wenli Feng, Chongqin Medical University, China. These cells were maintained in a RPMI-1640 medium containing 10% fetal bovine serum (FBS), 100 U/mL penicillin and 100 μg/mL streptomycin at 37°C in a 5% CO_2_ humidified atmosphere. Normal peripheral blood samples were obtained from three healthy donors. Peripheral blood samples were also obtained from three untreated chronic phase CML patients from the Department of Hematology, the Second People's Hospital of Guangdong province. Prior informed consent was obtained from the donors and patients with institutional board review approval.

### Xenograft mouse model of human CML

Balb/c nude mice were used for leukemogenesis experiments, and maintained in a temperature- and humidity-controlled environment. A total of 500,000 K562 cells transfected with RalA or empty vector were injected via intravenous tail vein into sublethally irradiated (3 Gy) Balb/c recipient mice. Imatinib (IM) was injected intraperitoneally at a dose of 100 mg/kg body weight for 10 consecutive days (10 mice per group; two independent experiments).

### Plasmid construction

To analyze the effects of RalA in human CML cells, K562 cells stably overexpressing RalA or control were established by transfecting K562 cells with RalA expression vector (RalA pEZ-M03) or empty vector (pEZ-M03) (Figure S2, S3). The cells were selected in medium containing G418 (700 μg/mL), and green fluorescent protein (GFP)-positive cells were finally sorted by fluorescence-activated cell-sorting (FACS) (BD Influx^™^, USA) (Figure S4). The expression of RalA was monitored by Western blot.

### G-LISA^™^ assay

Cells were lysed in lysis buffer in the presence of protease inhibitor cocktail. Cell lysates were analyzed with a microplate reader (Bio-Rad) for G-LISA. The RalA GTPase activity was measured by G-LISA™ Ral Activation Assay Biochem Kit™ (Cytoskeleton, Inc., Denver, CO, USA) according to the manufacturer's instructions. Briefly, lysates were incubated in a Ral-GTP affinity plate for 20 min followed by flicking out the solution from the wells and washing the wells twice with wash buffer at room temperature. Room temperature antigen presenting buffer was then added to each well using a multi-channel pipettor and incubated at room temperature for exactly 2 min. After flicking out the antigen presenting buffer, the wells were incubated with diluted anti-RalA primary antibody on an orbital microplate shaker (200–400 rpm) at room temperature for 45 min. After washing, the wells were incubated with diluted secondary antibody at room temperature for an additional 45 min. The wells were then incubated with horseradish peroxidase (HRP) detection reagent at room temperature for 15 min. HRP Stop Buffer was added to stop the reaction, followed by measurement of absorbance at 490 nm using a microplate spectrophotometer.

### miRNA and siRNA transfections

The sequences used in this study were: miR-181a mimic (sense: 5′-AACAUUCAACGCUGUCGGUGAG U-3′; antisense: 5′-UCACCGACAGCGUU GAAUGUU GU-3′); RalA siRNA (sense:5′-CGUGGAAACAUCUG CUAAATT-3′; antisense: 5′-UUUAGCAGAUGUUUCC ACGTA-3′). The RNA duplexes were synthesized and purified by Shanghai GenePharma Company (Shanghai, China), and stored at −20°C. All RNA duplexes (100 nM) were transfected into CML cells using Lipofectamine ™2000 according to the manufacturer's instructions.

### Real-time PCR assay

To measure RalA mRNA expression, total RNA was extracted from normal peripheral blood cells, CML cell lines, and CML patient samples using TRIzol (Invitrogen). After reverse transcription, the levels of RalA mRNA were determined using SYBR-Green real-time PCR assay. The PCR primers used were 5′-ATCGGAAGAAGGTAGTGC-3′ and 5′-AATCTGCTC CCTGAAGT-3′ (RalA); 5′-CAACGGATTTGGTCGTAT T-3′ and 5′-CACAGTCTTCTGGGTGGC-3′ (GAPDH). The levels of RalA mRNA were normalized to that of GAPDH and the fold-change was calculated using the 2^−ΔΔCT^ method.

### Western blot

K562 cells were lysed in RIPA buffer in the presence of proteinase inhibitor (Biocolor BioScience & Technology Company, Shanghai, China). Cell lysates (30 μg) were denatured in Laemmli sample buffer (Bio-Rad) for 5 min at 100°C, electrophoresed on 10% SDS–PAGE gel, and transferred to a polyvinylidene fluoride membrane. The membrane was blocked with 5% (w/v) fat-free milk in Tris-buffered saline (TBS) and 0.5% (v/v) Tween 20 for 1 h. The blots were then incubated with anti-RalA antibody (Santa Cruz Biotechnology, Inc.), anti-P38 MAPK antibody, anti-P-P38 MAPK antibody, anti-SAPK/JNK antibody, anti-P-SAPK/JNK antibody, and anti-GADPH antibody (Cell Signaling Technology, Danvers, MA, USA) at 4°C overnight. After washing, the blots were then incubated with HRP-conjugated secondary antibody. The signals were visualized with enhanced chemiluminescence (ECL) (BeyoECL Plus, Beyotime Company, Haimen, Jiangsu province, China) and analyzed using a UVITEC Alliance 4.7 gel imaging system (Cambridge, UK).

### MTT assay

Cell viability was determined by 3- (4, 5-dimethylthiazol-2-yl)-2, 4-diphenyl- tetrazolium bromide (MTT) assays. Briefly, K562 cells were seeded at a density of 1 × 10^5^ cells/mL in 96-well plates (100 μL/well). The cells were transfected with miR-181a mimic or siRNA (100 nM). After 6 h, the cells were treated with imatinib (IM). At 48 h post-treatment, 20 μL MTT stock solution (5 mg/mL) was added to each well, and the plate was incubated for 4 h at 37°C. The media was then removed, and dimethyl sulfoxide (DMSO) (150 μL) was added to dissolve the blue formazan crystals produced by live cells. Cell viability was assessed by measuring the absorbance at 570 nm on a Bio-Rad microtiter plate reader. RBC8, a selective RalA inhibitor, was purchased from Selleck Chemicals (Houston, TX, USA).

### Flow cytometry

FACS analysis was performed using a flow cytometer (BD FACSCalibur, San Jose USA). For cell viability analysis, cells were collected, rinsed twice with cold PBS, and then resuspended in 1 × Binding Buffer at a concentration of 1 × 10^6^ cells/mL. Cells were stained with 7-AAD by gentle vortexing and incubation at RT (25°C) for 15 min in the dark. Cells that stained positive for 7-AAD were either in the end stage of apoptosis, or undergoing necrosis.

### Cell migration and invasion assay

Cell migration was performed using the Transwell chamber assay (8-mm pore size, Corning, NY, USA). The Transwells were inserted into 24-well plates. Briefly, K562 cells (6 × 10^5^ cells, 200 μL) were seeded onto the upper chamber after transfection, while 600 μL RPMI-1640 medium with 20% FBS was added to the lower chamber. The upper chamber was removed after incubation at 37°C in a 5% CO_2_ humidified atmosphere for 24 hours, and cells which migrated to the lower chamber were counted under a microscope. The data were obtained from the average of the total number of migrated cells from triplicate experiments.

The invasiveness of K562 cells was analyzed using invasion chambers (24-well) coated with BD Matrigel™ matrix (8-mm pore size, BD Biocoat, Horsham, PA USA). Invasion was measured by determining the ability of K562 cells to migrate through Matrigel, a reconstituted basement membrane. A quantity of 600 μL RPMI-1640 with 20% FBS, which served as chemoattractant, was added to the lower compartment of the invasion chamber. K562 cells (3 × 10^5^) suspended in 100 μL serum-free RPMI-1640 were seeded in the upper compartment of the invasion chambers and incubated for 8 h. After 8 h, cells at the top of the BD Matrigel™ matrix insert (apical side) were removed by gently rubbing the area with a cotton swab moistened with medium. Invaded cells on the lower side of the membrane were fixed with methanol for 30 min and stained with hematoxylin for 5–10 min. Photomicrographs of five random fields were taken (magnification, × 100), and cells were counted to obtain the average number of cells that had invaded.

### Colony-forming assay

This was performed to measure the capacity of cell proliferation. After transfection with RalA expression plasmid, miR-181a mimic or RalA siRNA for 48 h, the cells (1 × 10^3^) were mixed completely with RPM-1640 medium containing 0.9% methylcellulose solution, 20% FBS, 2 mM L-glutamine, and 5 μM 2-mercaptoethanol, and seeded onto 24-well plates. Single cells were randomly and evenly distributed throughout the wells. Colonies were formed and counted 1 week later using an inverted microscope (Olympus, Japan). The number of colonies containing more than 50 cells was counted. All analyses were performed in triplicate.

### Phospho-specific protein microarray analysis

Phospho-array detection was performed in cooperation with Wayen Biotechnology (Shanghai, China). Cells transfected with RalA siRNA or miR-181a were collected 72 h post-transfection for the extraction of protein; 100 μg cell lysate was labeled with biotin reagent and hybridized on Phosphorylation ProArray (Full Moon BioSystems, CA, USA) using an Antibody Array Kit (Full Moon BioSystems) for the detection of 248-site-specific Cancer Signaling Phospho Antibody profiles. Finally, fluorescence intensity was scanned with a GenePix 4000B (Axon Instruments, Houston, TX, USA) using GenePix Pro 6.0. The raw data were treated via Grubbs' method. The phosphorylation ratio was calculated as follows: phosphorylation ratio = phospho value/unphospho value.

## SUPPLEMENTARY MATERIALS FIGURES AND TABLE


