# Supplementary material for: RalA, a GTPase targeted by miR-181a, promotes transformation and progression by activating the Ras-related signaling pathway in chronic myelogenous leukemia
Source: Oncotarget. 2016 Mar 8;7(15):20561–73. doi: 10.18632/oncotarget.7987 (PMC4991475; doi:10.18632/oncotarget.7987)
Supplement: Supplementary file 1 [file oncotarget-07-20561-s001.pdf]

RalA, a GTPase targeted by miR-181a, promotes transformation and progression by activating the Ras-related signaling pathway in chronic myelogenous leukemia

Supplementary Materials

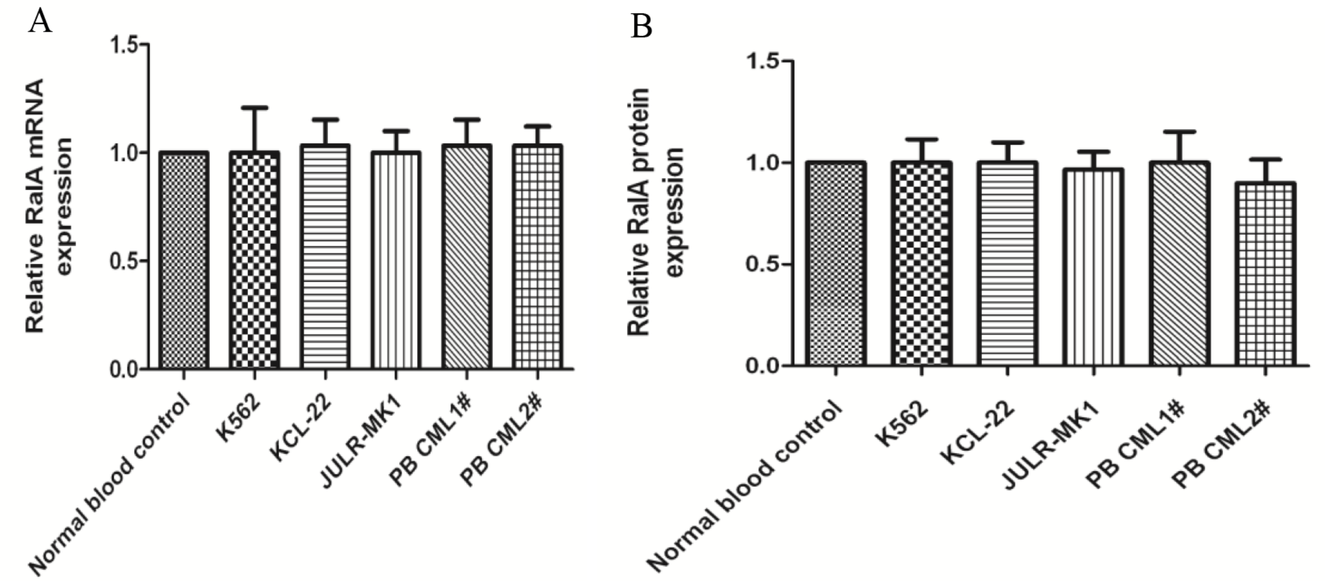

Supplementary Figure S1: Relative RalA expression in CML cells. (**A**) Real-time PCR assay of relative expression level of RalA mRNA in CML cells and samples. no groups exhibited significant changes ($P > 0.05$). (**B**) Western blot analysis of RalA protein of CML cells and samples. no groups exhibited significant changes ($P > 0.05$).

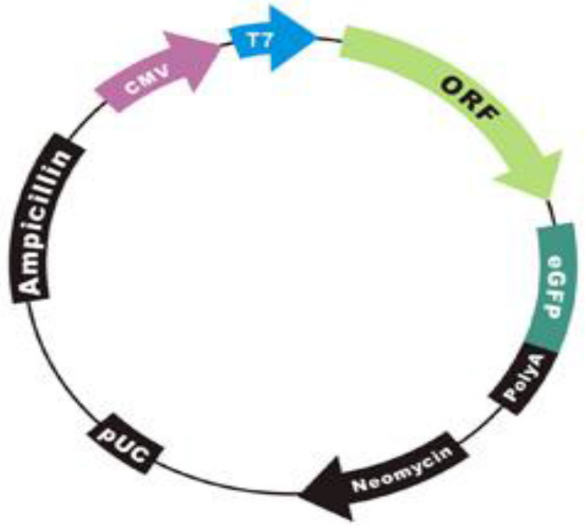

Vector Features

Promoter	CMV
Host Cell	Mammalian
Bacterial selection antibiotic	Ampicillin
Mammalian selection marker	Yes
Tag	C-eGFP

Supplementary Figure S2: Vector information for EX-B0058-M03. RalA was inserted into pEZ-M03 vector to form EX-B0058-M03.

```
  1 cggtaggcgt gtacggtggg aggtctatat aagcagagct ctctggctaa ctagagaacc
 61 cactgcttac tggcttatcg aaattaatac gactcactat agggagaccc aagctggcta
121 gttaagcttg atcaaacaag tttgtacaaa aaagcaggct tggaaggagt tcgaaccatg
181 gtcgactacc tagcaaataa gcccaagggt cagaattctt tggctttaca caaagtcatc
241 atggtgggca gtggtggcgt gggcaagtca gctctgactc tacagttcat gtacgatgag
301 tttgtggagg actatgagcc taccaaagca gacagctatc ggaagaaggt agtgctagat
361 ggggaggaag tccagatcga tatcttagat acagctgggc aggaggacta cgctgcaatt
421 agagacaact acttccgaag tggggagggg ttcctctgtg ttttctctat tacagaaatg
481 gaatcctttg cagctacagc tgacttcagg gagcagattt taagagtaaa agaagatgag
541 aatgttccat ttctactggt tggtaacaaa tcagatttag aagataaaag acaggtttct
601 gtagaagagg caaaaaacag agctgagcag tggaatgtta actacgtgga aacatctgct
661 aaaacacgag ctaatgttga caaggtattt tttgatttaa tgagagaaat tcgagcgaga
721 aagatggaag acagcaaaga aaagaatgga aaaaagaaga ggaaaagttt agccaagaga
781 atcagagaaa gatgctgcat tttatacctc gagtgcggcc gcaacccagc tttcttgtac
841 aaagtggttc gatctagaat ggctagcgtg agcaagggcg aggagctgtt caccggggtg
901 gtgcccatcc tgtcgagctg gacggcgacg taaacggcca caagttcagc gtgtccggcg
961 agggcgaggg cgatgcc
```

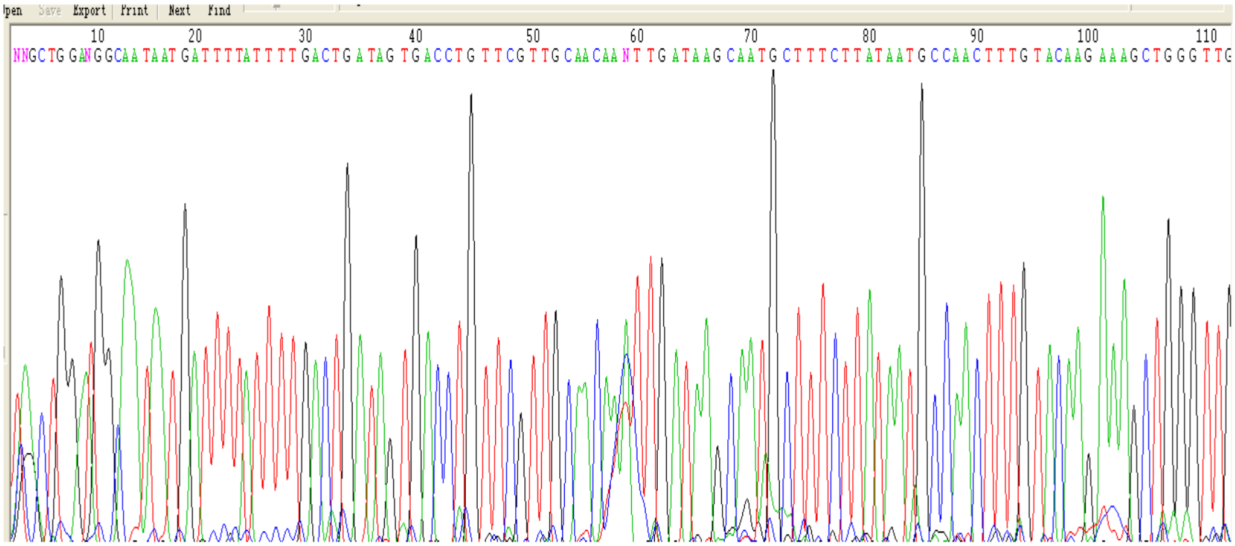

Supplementary Figure S3: The sequecing diagram of RalA plasmid (part).

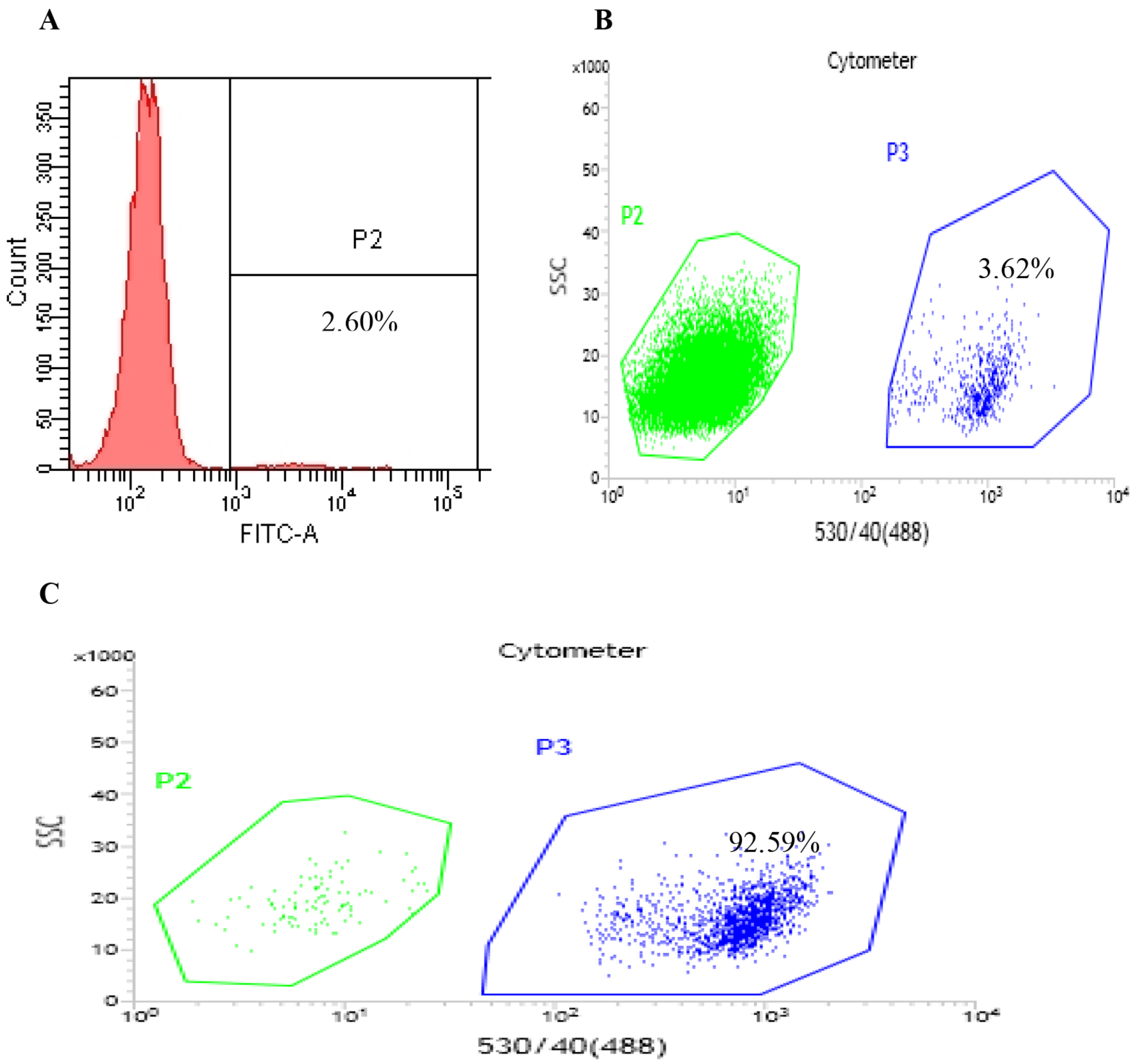

Supplementary Figure S4: FACS (BD Influx) sorting GFP positive K562 cells after transfection with RalA plasmid. (**A**) GFP rate of K562 cells transfected with RalA plasmid before sorted with FACS. K562 cells washed with cold PBS and GFP rate was detected with FACS , the rate was 2.60%. (**B**) GFP rate of K562 cells transfected with RalA plasmid when sorted with FACS. K562 cells were washed with cold PBS and GFP rate was detected when sorting with with FACS, the GFP rate was 3.62%. (**C**) GFP rate of K562 cells transfected with RalA plasmid after sorted with FACS. K562 cells were washed with cold PBS and GFP rate was detected after sorted with FACS, the GFP rate was 92.59%.

Supplementary Table S1: Phosphorylation signal pathway by integration of eight commonly downregulated phosphorylation signal molecules

KEGG PATHWAY	ID	Input Genes	*P*-Value
ErbB signaling pathway	hsa04012	SAPK/JNK Src P38 MAPK	0.00655529177188
GnRH signaling pathway	hsa04912	SAPK/JNK Src P38 MAPK	0.00760324811502
NOD-like receptor signaling pathway	hsa04621	SAPK/JNK P38 MAPK	0.0125608386305
TNF signaling pathway	hsa04668	SAPK/JNK P38 MAPK JunB	0.0136693571715
Colorectal cancer	hsa05210	SAPK/JNK P38 MAPK	0.0155731338224
VEGF signaling pathway	hsa04370	Src VEGFR2 P38 MAPK	0.0162200972063
Pancreatic cancer	hsa05212	SAPK/JNK P38 MAPK	0.0182506095252
Fc epsilon RI signaling pathway	hsa04664	SAPK/JNK P38 MAPK	0.0211678802873
Osteoclast differentiation	hsa04380	SAPK/JNK P38 MAPK JunB	0.0261746365106
Focal adhesion	hsa04510	SAPK/JNK P38 MAPK Src VEGFR2	0.0287905207274
Toll-like receptor signaling pathway	hsa04620	SAPK/JNK P38 MAPK	0.0606718992031
Neurotrophin signaling pathway	hsa04722	SAPK/JNK P38 MAPK	0.0773124843557
Acute myeloid leukemia	hsa05221	P38 MAPK c-Kit	0.0819213197905
Adipocytokine signaling pathway	hsa04920	SAPK/JNK	0.11715504168
Insulin signaling pathway	hsa04910	SAPK/JNK P38 MAPK	0.112289072507
RIG-I-like receptor signaling pathway	hsa04622	SAPK/JNK	0.11715504168
Thyroid cancer	hsa05216	P38 MAPK	0.215520758417
T cell receptor signaling pathway	hsa04660	SAPK/JNK P38 MAPK	0.216151824228
Wnt signaling pathway	hsa04310	SAPK/JNK	0.320210291548
MAPK signaling pathway	hsa04010	SAPK/JNK P38 MAPK	0.355531741604
mTOR signaling pathway	hsa04150	P38 MAPK	0.389762952625
Pathways in cancer	hsa05200	SAPK/JNK P38 MAPK	0.277474423054
Glioma	hsa05214	P38 MAPK	0.41400998985
Chronic myeloid leukemia	hsa05220	P38 MAPK	0.450827803876
B cell receptor signaling pathway	hsa04662	P38 MAPK	0.455265208431
TGF-beta signaling pathway	hsa04350	P38 MAPK	0.481150746969
Hematopoietic cell lineage	hsa04640	c-Kit	0.513771561689
PI3K-Akt signaling pathway	hsa04151	VEGFR2 P38 MAPK c-Kit	0.537295853734
MicroRNAs in cancer	hsa05206	P38 MAPK	0.910873982664
